# Detection of a Bocavirus Circular Genome in Fecal Specimens from Children with Acute Diarrhea in Beijing, China

**DOI:** 10.1371/journal.pone.0048980

**Published:** 2012-11-02

**Authors:** Hui Zhao, Linqing Zhao, Yu Sun, Yuan Qian, Liying Liu, Liping Jia, You Zhang, Huijin Dong

**Affiliations:** 1 Graduate School of Peking Union Medical College, Beijing, China; 2 Laboratory of Virology, Capital Institute of Pediatrics, Beijing, China; Columbia University, United States of America

## Abstract

To determine if human bocavirus 2 (HBoV2) has a circular genome similar to the head-to-tail sequence of HBoV1 and the episomal form of HBoV3, 15 HBoV2 positive samples identified from 553 stool specimens from children with acute diarrhea were tested for a head-to-tail sequence using TaqMan-based real-time PCR. A circular genome with a head-to-tail sequence was identified in one (BJQ435) out of 15 samples tested by nested PCR. The complete circular genome of HBoV2-C1 (BJQ435) was 5307 nt in length and was flanked with a 520 nt-long terminal non-coding region (NCR). The secondary structure of HBoV2 -C1 had some differences compared to HBoV3-E1 (JN086998). Our study indicates that the HBoV genome exists in the form of a head-to-tail monomer and provides more information for understanding the HBoV replication mechanism.

## Introduction

Since human bocavirus (HBoV) was first discovered in respiratory secretions by Allander *et al*. in 2005 [1], three additional genotypes of HBoV have been reported in succession: HBoV2, HBoV3, and HBoV4 [2]–[4]. These HBoVs were classified into the Bocavirus genus (family *Parvoviridae*, subfamily *Parvovirinae*) and considered to be the second parvovirus discovered to date, besides parvovirus B19, which can cause infectious diseases in human beings.

Accumulating clinical data in recent years have indicated that HBoV1 is an important pathogen in lower respiratory tract illnesses, but very little is known about other human HBoVs [5]. HBoVs 2–4 are mainly detected in stool samples, but have also been found in nasopharyngeal specimens [6], [7]. The high detection rate and high degree of genetic diversity in these enteric viruses from stool specimens (especially for HBoV2) suggest that they may be pathogenic viruses in acute gastroenteritis, although current data show contradictory conclusions [2], [4], [8]. Although HBoV1 has been successfully cultured in primary differentiated human airway epithelial cells [9], the pathogenesis of HBoVs is not well understood due to a lack of a simplified culture system or animal model.

HBoVs are minute, non-enveloped viruses containing single-stranded DNA of approximately 5 kb in length. In contrast to other parvoviruses, HBoV has a third open reading frame (ORF) in the middle of the genome that encodes nuclear phosphoprotein NP1, and the other two ORFs putatively encode nonstructural protein NS1 and capsid proteins VP1 and VP2 [9], [10]. All parvoviruses of vertebrates have similar genome structures with terminal repeats required for DNA replication, and many of them have different palindromes at each end serving as primers for DNA replication, which occurs through a single-strand displacement mechanism, called rolling hairpin replication. Head-to-tail sequences of HBoV1 were detected in the supernatants of cell culture and in HBoV1-positive clinical respiratory specimens, suggesting that the HBoV uses rolling circle replication rather than rolling hairpin replication, which is the general mechanism for parvoviruses [11]. At approximately the same time, another study detected the head-to-tail sequence in an episomal circular form of the genome for HBoV3 from an intestinal biopsy of a child with gastrointestinal symptoms [12].

In this study, we report the first circular genome of HBoV2 detected in a stool specimen from a child with acute diarrhea, which could provide a greater understanding of the HBoV replication mechanism and its role in enteric infection.

## Materials and Methods

### Ethics Statement

This project was approved by the ethics committee of the Capital Institute of Pediatrics. This project did not involve personal data of the patients, such as name, age, and gender. In addition, the stool samples used for this study were left over after routine laboratory testing of children with diarrhea treated in the outpatient department, and therefore additional physical and financial burdens were not incurred by the patients. Verbal informed consent was obtained from parents or guardians, which was approved by the ethics committee of the Capital Institute of Pediatrics. Verbal consent was administered by two staff members involved with the study (clinician or researcher) and was obtained. The registration form was completed by signatures from the staff members.

### Clinical samples

Five hundred and fifty three stool samples were collected from pediatric patients (351 males and 202 females) who were diagnosed with acute diarrhea in the outpatient department of the Affiliated Children's Hospital of the Capital Institute of Pediatrics from November 2010 to October 2011. The age of the patients ranged from 35 days to 15 years (mean: 15 months). Acute diarrhea was defined as a condition where the patient experienced three or more loose or liquid stools per day for less than 2 weeks. All stool samples were diluted 1∶10 in phosphate buffer, vortexed, and then centrifuged at 4,000 rpm for 15 min. The supernatants were collected in sterile tubes and stored at −20°C before use.

### Nucleic acid extraction

DNA was extracted from the supernatants of stool samples using DNAzol® BD (Molecular Research Center, USA) following the manufacturer's instructions. The extracted DNA was re-suspended in 20 μl of 8 mM NaOH.

### Detection of HBoV2

All of the samples were tested for HBoV2 DNA by TaqMan-based real-time polymerase chain reaction (PCR) targeting the conserved sequence of the HBoV2 nonstructural gene NS1. The primers and probe were designed based on the sequences of HBoV2 GU301645 from GenBank as follows (5′ to 3′): TTGCTCCTGGGACTGAACGT for forward primer HBoV2F, TTCCCTGACAG GATCATCTTC for reverse primer HBoV2R, and FAM-TCATGATCCAACTAAGAAACTGCGCACCA -BHQ1 for probe HBoV2P. Each reaction contained 2.5 μl DNA, 0.5 μl of each of forward and reverse primers (40 μM), 0.5 μl probe (10 μM), 12.5 μl 2× TaqMan Universal Master Mix (Applied Biosystems, USA), and diethyl-pyrocarbonate (DEPC)-treated water for a final volume of 25 μl. The thermal cycling program consisted of 50°C for 2 min and 95°C for 10 min followed by 40 cycles of 95°C for 15 s and 60°C for 1 min. The pGEM-T NS1 HBoV2 plasmid (1×10^4^ copies) was included in each run as a positive control, which had been previously constructed in our laboratory, and DEPC-treated water was included as a negative control for the standard curve. A control of phosphate buffer was also included in the nucleic acid extraction and then amplified in each run.

### Amplification of HBoV2 gene fragments and circular genome sequencing

The sequences of HBoV2, except for the unknown termini, were obtained from stool samples that were positive for HBoV2 DNA by segmented amplification using the different primers shown in Table 1. DNA (2 μl of each) extracted from stool samples was mixed with 12.5 μl 2×GreenMix (Promega, USA), 0.5 μl of the forward and reverse primers (10 μM) each, and 9.5 μl DNAase/RNAase free water in a total volume of 25 μl. The thermal cycling program for the *VP2* gene using primers VP2-S-F and VP2-S-R was 94°C for 5 min, followed by 45 cycles at 94°C for 45 s, 48°C for 45 s, and 72°C for 30 s, and a final extension at 72°C for 7 min. The thermal cycling program for the *VP2* gene with primers VP2F1 and VP2R1 was similar to that with the VP2-S-F and VP2-S-R primers, except that the extension time was 1 min 30 s instead of 30 s. All other PCRs were performed with the following temperature condition: 94°C for 5 min, followed by 45 cycles at 94°C for 45 s, 50°C for 45 s, and 72°C for 1 min 30 s, and a final extension at 72°C for 7 min. PCR products of the expected size were purified using an EasyPure Quick Gel extraction kit (TransGen Biotech, Beijing, China) and then inserted into the pGEM-T vector using a DNA ligation kit (Promega, USA). Then recombinant DNAs were transformed into the *Escherichia coli* strain DH5α for sequencing (Invitrogen, Beijing, China) from both directions.

**Table 1:** Primers for amplification of the HBoV2 genome.

Target genes	Primers	Position	Sequences (5′–3′)	Length (nt)
NS1	HBoV2F	GU301645:197–216	ttgctcctgggactgaacgt	77
	HBoV2R	GU301645:253–273	ttccctgacaggatcatcttc	
	HBoV2P	GU301645:218–246	tcatgatccaactaagaaactgcgcacca	
NS1	NS1F1	GU048663:1–16	tgccggcagacatatt	
	NS1R1	GU048663:901–918	aggagagatcaaccgatt	918
	NS1F2	GU048663:822–841	atacagagacaagcgaggtg	
	NS1R2	GU048663:1648–1665	taaacactcctcccacca	844
	NS1F3	GU048663:1514–1531	gcttttatggtcctgctt	
	NS1R3	GU048663:2293–2310	tctcttcttggatggacg	797
NP1	NP1F	GU048663:2291–2308	atcgtccatccaagaaga	
	NP1R	GU048663:2907–3014	aaagcatttcttcgtctg	723
VP1	VP1F	GU048663:2935–2952	ttgggatgatgtctaccg	
	VP1R	GU048663:3519–3536	acccacaccagaaccttt	602
VP2	VP2F1	GU048663:3397–3414	ataacgagcctaaaccag	
	VP2R1	GU048663:4167–4184	aatgtatgctctttcgtt	788
	VP2-S-F	FJ170278:3954–3971	gaagacgcaaatgctgta	
	VP2-S-R	FJ170278:4162–4179	ctgtgtttccgtgctgtc	226
	VP2F2	FJ170278:4162–4179	gacagcacggaaacacag	
	VP2R2	FJ170278:5179–5196	atgcctgacgcagtacaa	1035
Termini	HBoV2-F1		gaagggtgactgtaatcc	
	HBoV2-R1		gctccatagtaagtgctc	unknown
	HBoV2-F2		tacagtccgatggcagtg	
	HBoV2-R2		agtctgacgagatgcgga	unknown

All stool samples positive for HBoV2 were detected for head-to-tail sequences using a nested PCR. Based on the sequences of HBoV2 known from the step described above, primers for the terminal sequences were designed as shown in Table 1. Forward primers were designed according to the 3′end sequence and reverse primers were designed according to the 5′ end sequence. The components of the PCR reaction were similar to those described in the previous step. Primers HBoV2-F1 and HBoV2-R1 were used for the first round of PCR, and HBoV2-F2 and HBoV2-R2 were used for the second round. The first round of the PCR reaction was performed according to the protocol as follows: initial denaturation at 94°C for 5 min, followed by 45 cycles at 94°C for 45 s, 49°C for 45 s, and 72°C for 1.5 min, and a final extension at 72°C for 7 min. The protocol for the second round of PCR was similar to that of the first round, except that the annealing temperature was 51°C instead of 49°C. The PCR products were purified, cloned, and sequenced as described for the previous step.

All of the DNA fragments were assembled by using SeqMan to obtain the complete HBoV2 circular genome (HBoV2-C1) with a head-to-tail sequence, which was submitted to GenBank to obtain the accession number (JX257046).

### Sequence analysis for genomic DNA and prediction for the secondary structure

To determine the sequence relationship of HBoV2-C1 with other known HBoVs, genomic DNA sequences of HBoVs from different regions of the world and the representative sequences of HBoV2A and HBoV2B [2] were chosen from GenBank for sequence analysis. These sequences were AB481072, DQ000495, DQ988933, FJ695472, and HQ585888 for HBoV1; EU082213, FJ170278, FJ375129, FJ948860, FJ973558, FJ973559, FJ973560, GQ200737, and GU048663 for HBoV2; EU918736, FJ973562, JN086998, and GQ867666 for HBoV3; and FJ973561 and NC_012729 for HBoV4 (Table 2). The nucleotide sequences of HBoV2-C1 were first aligned with the sequences listed above using ClustalW. Maximum likelihood (ML) phylogenetic trees were then constructed using the model of General Time Reversible (GTR) and discrete Gamma distribution (+G) with 5 rate categories in Mega 5. The mfold web server (http://mfold.rit.albany.edu/?q=mfold/DNA-Folding-Form) was used to predict the secondary structure of the non-coding region (NCR) containing the head-to-tail sequence, which was located between the VP1/VP2 and NS1 genes of HBoV2-C1 and HBoV3-E1 (JN086998).

**Table 2:** Genomic DNA used for sequence analysis.

Genotype	GenBank No.	Location
HBoV1	AB481072	Japan
	DQ000495	Sweden
	DQ988933	China
	FJ695472	Germany
	HQ585888	Tunisia
HBoV2	FJ375129	China
	FJ948860	Australia
	GQ200737	Pakistan
	GU048663	Thailand
HBoV2A	EU082213	Australia
	FJ170278	Pakistan
	FJ973558	Tunisia
HBoV2B	FJ973559	Nigeria
	FJ973560	Nigeria
HBoV3	EU918736	Australia
	FJ973562	Tunisia
	JN086998	America
	GQ867666	Brazil
HBoV4	FJ973561	Nigeria
	NC_012729	Nigeria

## Results

### Prevalence of HBoV2 in children with acute diarrhea

Out of the 553 specimens tested, 15 (15/553; 2.7%) were HBoV2 DNA positive. Among the 15 positive specimens, 5 were positive with cycle threshold (Ct) values less than 30 and the other 10 specimens were positive with higher Ct values (≥30).

### Detection of head-to-tail sequence of HBoV2 in stool samples

**Figure 1:**
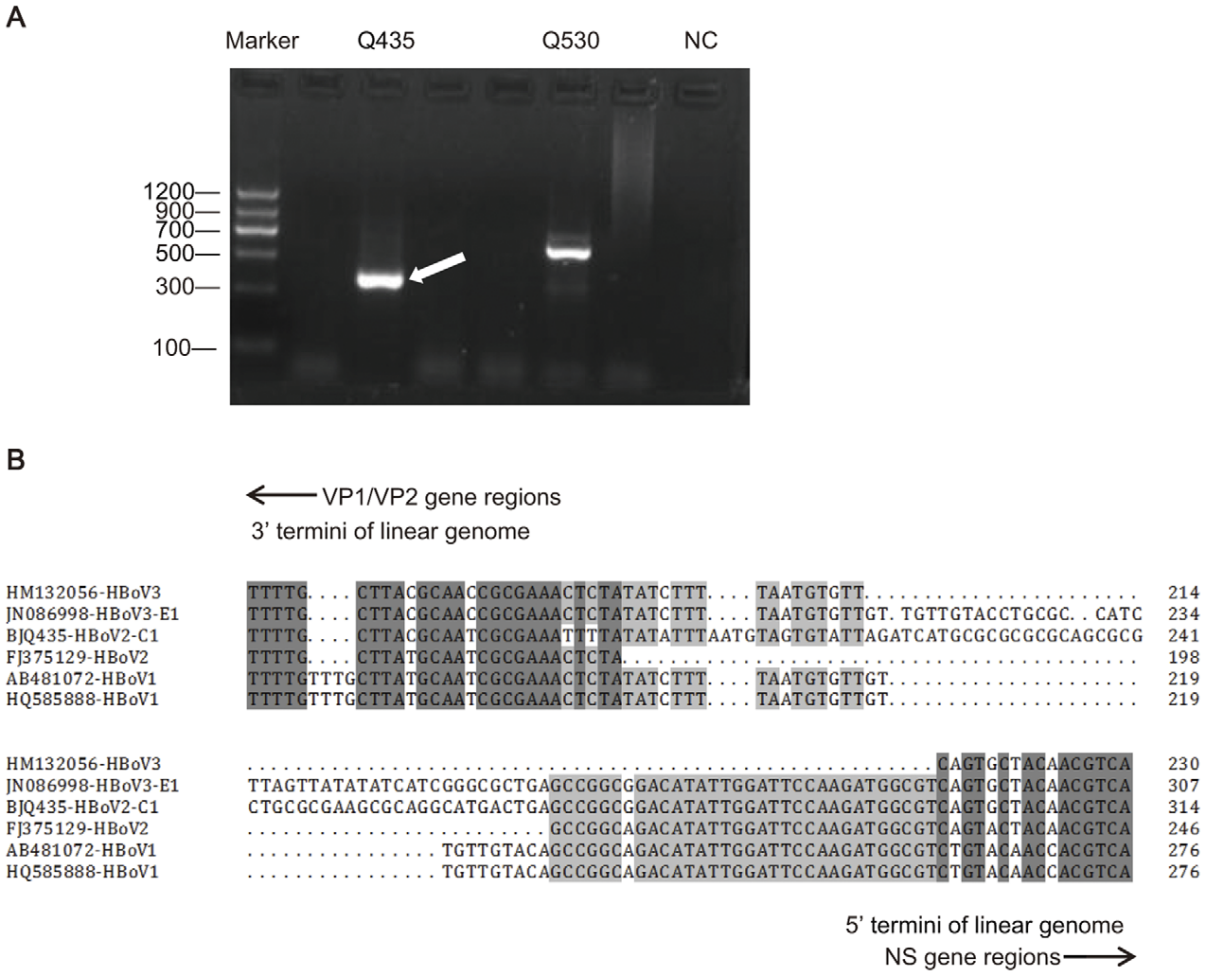
Products of nested PCR and nucleotide alignment of HBoV genomic termini. (A): The results from nested PCR of 6 HBoV2-positive samples resolved by agarose gel electrophoresis with a DNA molecular weight marker and negative control (NC). The arrow indicates the positive sample by nested PCR, which was further identified as HBoV2 by DNA sequencing. (B): Alignment of known terminal genome of HBoV1, 2, and 3, in which the genome of HBoV2-C1 and JN086998-HBoV3-E1 is circular. The nucleotide number is counted according to the sequence of the non-coding regions by connecting 5′ and 3′ termini.

To assess the head-to-tail sequence of HBoV2, we next tested all 15 HBoV2 DNA positive samples that had been confirmed by *VP1* sequencing (data not shown) using nested PCR. However, only two samples had PCR products with the expected size, and one of them (BJQ435) was confirmed as positive for the head-to-tail sequence of HBoV2 by sequencing (Fig. 1A). Its circular genome was labeled as HBoV2-C1 (GenBank accession no. JX257046).

### Characterization of the HBoV2 circular genome

**Figure 2:**
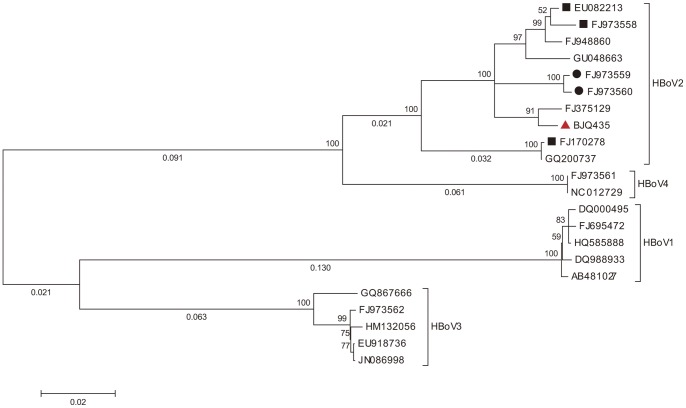
Phylogenetic analysis of HBoV2-C1 (BJQ435, red triangle) and other nearly complete genomes of HBoV1-4. Nucleotide alignments were performed using the genomic sequences, except for the terminal non-coding region between the *VP1/VP2* and *NS1* genes. A phylogenetic tree was constructed using the maximum likelihood method with the GTR + G model and 1,000 bootstrap replicates in MEGA 5. The percent bootstrap higher than 50 is shown at nodes, and branch lengths more than 0.02 are shown below the branches. Reference strains of HBoV2A and HBoV2B were labeled with black rectangles and black circles, respectively.

The complete circular genome of HBoV2-C1 (BJQ435) was 5307 nt in length. A search of the ORF in NCBI identified four ORFs for HBoV2-C1, which consisted of 1923 nt for NS1, 648 nt for NP1, 2004 nt for VP1, and 1617 nt for VP2 encoding four proteins of 640, 215, 667, and 538 amino acids in length, respectively. These four proposed ORFs were flanked with a 520 nucleotide long NCR between the *VP1/VP2* and *NS1* genes. Sequence analysis revealed that HBoV2-C1 shared high nucleotide sequence homology (>98%) with FJ375129, which was an incomplete genome found in Shanghai, China and was closer to HBoV2B (Fig. 2).

### Sequence analysis and prediction for the secondary structure of HBoV2-C1 termini

Alignment of HBoV1-3 termini showed that the NCR of HBoV2-C1 shared high nucleotide sequence identity with that of HBoV3-E1 (90.8%), and there were approximately 50 newly detected nucleotides linking the head to tail in HBoV2-C1, which had not been found in previously reported HBoV2 genomic DNA sequences. The most variable region with less than 50% sequence identity between HBoV2-C1 and HBoV3-E1 was found in the middle of the 50 nucleotides (Fig. 1B).

Prediction for the secondary structures of HBoV2-C1 and HBoV3-E1 showed that both of them had two major hairpin structures called “hairpin-1” and “hairpin-2”, respectively, and a cluster of stems and loops mainly located in the 5′ upstream region and closer to the *NS1* gene, which is called the “5′ terminus structure” [12]. Compared to HBoV3-E1, HBoV2-C1 had a more complicated hairpin-2 with an additional rabbit ear-like structure (consisting of structures 8 and 9). The second major difference was that the stems and loops in the 5′ terminus structure were more scattered in HBoV2-C1 than in HBoV3-E1 (Fig. 3).

**Figure:**
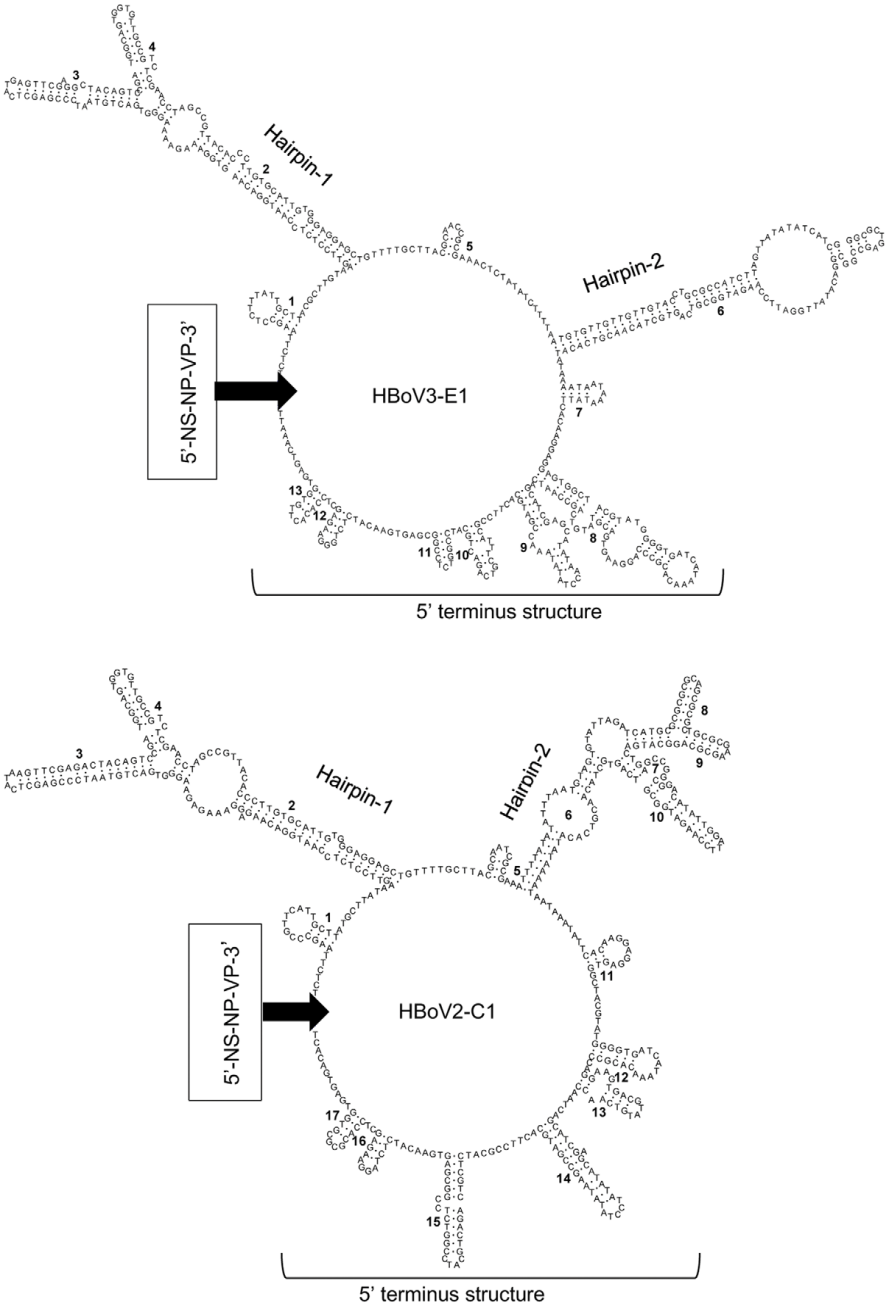
Secondary structure prediction and sequences for the non-coding region of HBoV3-E1 and HBoV2-C1. The arrow indicates the location of the region containing the *NS*, *NP*, and *VP* genes (black rectangle), with the exception of the non-coding region, oriented 5′ to 3′.

## Discussion

It is well known that many autonomous parvovirus genomes have different palindromes at each end and mainly package the minus DNA strand, while dependoviruses, such as the adeno-associated virus (AAV), can efficiently replicate in cells with the assistance of helper adenoviruses or herpesviruses in most cases. However, in the absence of helper virus, AAVs establish latent infection in an extra-chromosomal concatemer state or by chromosomal integration. HBoV was considered to be an autonomous human parvovirus with a single strand genome that was predominantly packaged as a minus DNA strand [13]. In addition, the successful propagation of HBoV1 in pseudo-stratified human airway epithelium [9] and replication of a full-length HBoV1 genome cloned into a plasmid in the absence of helper virus [14] further confirmed the finding that HBoV can replicate autonomously. However, due to the frequent occurrence of simultaneous co-infections with other viruses, it is unknown whether other viruses work as helper viruses for HBoV. One case report describing the co-infection of HBoV1 and human herpes virus type 6 (HHV6) as well as a fatal case of an immune-competent child with pneumonia caused by co-infection with adenovirus type 7 and HBoV are in agreement with the hypothesis that HBoV infection may depend on helper viruses under clinical conditions, which may in turn change the HBoV replication mechanism [15], [16].

Recently, Lüsebrink *et al.* used self-priming elongation assays to test for replication intermediates that were head-to-head and tail-to-tail structures in rolling hairpin replication, and head-to-tail sequences of HBoV1 were surprisingly detected instead [11]. Kapoor *et* *al*. also reported the presence of the extra-chromosomal circular episomal form of HBoV3 using nested inverse PCR assays [12]. These results support the hypothesis that HBoVs may establish latent infection in an episomal form instead of forming concatemers. Meanwhile, the detection of a circular genome of HBoV2 in this study further provided evidence for this hypothesis. These findings are in agreement with the hypothesis that the replication model of HBoV could be rolling circle replication, because head-to-tail sequences in viral genomes are typical features of rolling circle replication rather than rolling hairpin replication adopted by other parvoviruses, such as the minute virus of mice (MVM) [15], [17]. However, rolling circle replication is not a proven model for HBoVs [18]. A recent study reported that the terminal hairpins of the HBoV1 genome from a nasopharyngeal aspirate of an infected patient were identified and a reverse genetics system was established based on the full-length genome, which strongly supports the hypothesis that HBoV should follow rolling hairpin replication [14]. Taken together, these findings suggest that head-to-tail junctions may be compatible with rolling hairpin replication, and that they were likely generated during hairpin-dependent replication [19]. Since a similar head-to-tail sequence was also detected in a novel porcine parvovirus, PPV4 [20], and the majority of AAV DNA can persist mainly as circular episomes in human tissues [21], the episomal structures of HBoV1∼3 could be the storage forms of persistent infection in human tissues.

To the best of our knowledge, this is the first report identifying the circular genome of HBoV2 in clinical samples. Due to the novel linker sequence of HBoV1 that shares high similarity to parts of the terminal hairpins of the bovine parvovirus 1 (BPV1) and the minute virus of canines (MVC) [11], Oliver Schildgen *et al*. [18] hypothesized that HBoV1 terminal repeats are hybrid relics of HBoV's predecessors (BPV1 on the left-hand side and MVC on the right-hand side), and therefore compared the terminal sequences of BPV1, MVC, HBoV1, and HBoV3. The new terminal sequence in HBoV3 was shorter than that in HBoV1, and the sequences identical to BPV1 (3′-CGCGCGTA-5′and 3′-GATTAG-5′) and MVC (5′-GCGCCTTAGTTATATATAACAT-3′) of HBoV1 were missing in HBoV3. Similar observations were made in HBoV2-C1 from this study (data not shown), and the newly detected sequence of HBoV2-C1 shared low sequence identity (<50%) with that of HBoV3-E1, although the complete NCRs shared a high sequence identity (>90%) with it.

These head-to-tail sequences have been previously elucidated by Lüsebrink *et al*. in detail [11], and they may be products of recombination or a novel feature in the replication cycle of HBoVs or part of a dead-end replication product. Regardless of how they are generated, the high sequence homology and similar secondary structure suggests that the terminal sequences might play an important role in the replication of HBoVs in the evolution process. The low sequence identity between the new 50 nt-long sequences detected may be due to different tissue tropisms of the various HBoVs, since the detection rate of HBoV2 was highest among the enteric species [2]. It is well known that the AAV genome always integrates into human chromosome 19 in the absence of helper virus, but the absence of targeted integration does not preclude the possibility of random integration into the host genome [21]. The head-to-tail AAV DNA was inserted into the host genome as a provirus with deletions and extensive rearrangements in the inverted terminal repeat sequences [22]. Although there is no evidence of HBoV DNA integration into the host genome reported to date, we cannot rule out the possibility that HBoV integrates into a rare minority infected cells [12], which may contribute to the variety of HBoV terminal sequences observed. Therefore, another possibility is that the sequence is inserted in infected cells, and a completely as yet unknown replication mechanism occurs that is atypical for parvoviruses [11].

Out of the 15 HBoV2-positive stool samples, the circular genome was only detected in one sample (BJQ435) with a lower Ct value in real-time PCR, suggesting that there may be more chances to detect the head-to-tail sequence in samples with higher virus load. Therefore, future experiments are needed to confirm the terminal sequence from more HBoV2-positive stool samples, and especially the newly detected head-to-tail sequence. Two other pairs of primers (forward-head and reverse-tail primers) were used for detecting the positive sense strand (data not shown), but unfortunately no product was detected. These results support the hypothesis that HBoV genomes mainly exist in minus strands, which is in consistent with previous findings [11], [13]. Another limitation of this study was that the number of stool samples was insufficient for detecting the chromosome-integrated form of HBoV2, and therefore additional studies with a larger number of samples are needed.

Our detection of a circular genome of HBoV2 in stool samples provides a deeper understanding of the replication mechanism of HBoV2, and may make it possible to rescue the HBoV2 genome in future studies. Due to a higher prevalence of HBoV2 in acute gastroenteritis than in respiratory infection, the data from this study are also helpful for determining whether HBoV2 plays a role in enteric diseases.
